# Zinc and Oxidative Stress: Current Mechanisms

**DOI:** 10.3390/antiox6020024

**Published:** 2017-03-29

**Authors:** Dilina do Nascimento Marreiro, Kyria Jayanne Clímaco Cruz, Jennifer Beatriz Silva Morais, Jéssica Batista Beserra, Juliana Soares Severo, Ana Raquel Soares de Oliveira

**Affiliations:** Department of Nutrition, Federal University of Piauí, Campus Minister Petrônio Portela, Teresina 64049-550, Brazil; kyriajayanne@hotmail.com (K.J.C.C.); jenniferbeatriz.morais@gmail.com (J.B.S.M.); jessica_beserra@hotmail.com (J.B.B.); ju_ssevero@hotmail.com (J.S.S.); ana_luizamo@hotmail.com (A.R.S.d.O.)

**Keywords:** zinc, oxidative stress, mechanisms

## Abstract

Oxidative stress is a metabolic dysfunction that favors the oxidation of biomolecules, contributing to the oxidative damage of cells and tissues. This consequently contributes to the development of several chronic diseases. In particular, zinc is one of the most relevant minerals to human health, because of its antioxidant properties. This review aims to provide updated information about the mechanisms involved in the protective role of zinc against oxidative stress. Zinc acts as a co-factor for important enzymes involved in the proper functioning of the antioxidant defense system. In addition, zinc protects cells against oxidative damage, acts in the stabilization of membranes and inhibits the enzyme nicotinamide adenine dinucleotide phosphate oxidase (NADPH-Oxidase). Zinc also induces the synthesis of metallothioneins, which are proteins effective in reducing hydroxyl radicals and sequestering reactive oxygen species (ROS) produced in stressful situations, such as in type 2 diabetes, obesity and cancer. Literature provides strong evidence for the role of zinc in the protection against oxidative stress in several diseases.

## 1. Introduction

Oxidative stress is characterized by an imbalance between oxidants and antioxidants, due to the excessive production of reactive oxygen species (ROS) and the reduction in the rate of its removal by the antioxidant defense system. This metabolic disturbance favors the oxidation of biomolecules, contributing to the oxidative damage in the cells and tissues and consequently to the development of several chronic diseases such as obesity, diabetes and cancer [1,2,3].

Several studies have examined the role of minerals in the antioxidant defense system. In particular, zinc is one of the essential minerals for human health because it serves as a co-factor for over 300 enzymes and 2000 transcription factors. Zinc is an important mediator of cellular signaling [4,5]. It acts primarily to enhance insulin action. As an anti-inflammatory agent, zinc provides structural stability to cell membranes and it is also an important regulator of gene expression [6,7,8].

Zinc acts as a co-factor for important enzymes that contribute to the proper functioning of the antioxidant defense system. In addition, this mineral protects cells against oxidative damage because it acts in the stabilization of membranes, inhibits the enzyme nicotinamide adenine dinucleotide phosphate oxidase (NADPH-Oxidase), a pro-oxidant enzyme, and induces metallothionein synthesis. Metallothionein is involved in the reduction of hydroxyl radicals (OH) and in the sequestration of the reactive oxygen species produced under stress conditions [9,10].

Therefore, considering the complexity of oxidative stress and its detrimental effect on health in addition to the potent antioxidant properties of zinc, this review aims to provide the latest information on the mechanisms involved in the role of zinc in the protection against oxidative stress in chronic diseases.

This is a narrative review and a bibliographical survey of articles in the databases PubMed and Science Direct without limit for the year of publication, selected from August to September 2016. The keywords used in the search were "zinc”, “oxidative stress”, “zinc transporters”, and “antioxidant”. The descriptors were used alone or combined using the Boolean operators “AND” and “OR”.

Studies that presented relevant aspects of the mechanisms involved in the role of zinc in protection against oxidative stress were included. Dissertations, theses, articles in which only a summary was available, and those duplicated in different databases were excluded.

Then, we proceeded with the analysis of the included articles, and started reading the titles, followed by summaries, and later the full text. The application of the exclusion criteria was performed at all stages, always by consensus of the reviewers. At the end, we selected 63 articles.

## 2. Biochemical Aspects

The role of zinc in the antioxidant defense system has been widely investigated. Studies have highlighted its role in the regulation of glutathione peroxidase and in the expression of metallothionein, as well as its role as a co-factor for superoxide dismutase. Moreover, zinc competes with iron and copper in the cell membrane, inhibits the NADPH-oxidase enzyme, and reduces chronic inflammation and hyperglycemia [11,12].

Zinc is a structural component of the enzyme superoxide dismutase present in the cytoplasm of cells. Superoxide dismutase has an active center with a copper ion and a zinc ion. This enzyme promotes the conversion of two superoxide radicals to hydrogen peroxide and molecular oxygen, reducing the toxicity of ROS because it converts a highly reactive species to a less harmful one [13].

Therefore, maintaining adequate concentrations of zinc in the cell compartments is essential for the proper functioning of the antioxidant defense system. Examining this function, Homma et al. [14] found that zinc deficiency appears to induce a mutant-like conformation in superoxide dismutase that induces chronic endoplasmic reticulum stress. Consequently, this results in the inhibition of protein synthesis and induction of zinc transporter Zip-14.

Another mechanism by which zinc acts as an antioxidant is by affecting the expression of glutamate-cysteine ligase, which is the rate-limiting enzyme of glutathione de novo synthesis. This has a two-fold effect of zinc to neutralize free radicals directly by glutathione or indirectly as a glutathione peroxidase cofactor [15]. Ha et al. [16] showed that the administration of 100–150 mM of zinc in the cultured human retinal pigment epithelial cell line ARPE-19 cells upregulates the mRNA levels of glutamate-cysteine ligase via an nuclear factor erythroid 2 (NFE2)-related factor 2 (Nrf2)-dependent pathway. In this way, zinc modulates the total cellular glutathione concentration [17].

Zinc is a potent metallothionein inducer. Zinc is bound to metallothionein under normal physiological conditions. In oxidative stress conditions, the micronutrient is released from its complex with metallothionein and is redistributed in the cells to exert antioxidant actions [18,19]. Liang et al. [20] observed increased metallothionein expression in the liver of rats receiving zinc supplementation (5 mg/kg of body weight per day), which favored antioxidant and anti-inflammatory effects mediated by metallothionein.

It is noteworthy that metallothionein has the ability to effectively bind heavy metal ions, such as zinc, copper, chromium, cadmium, mercury [21,22,23]. Therefore, this enzyme plays a significant role in cell protection against excessive amounts of metal ions, thus functioning as the main system of detoxification against toxic heavy metals. In this way, metallothionein acts against oxidative stress [24].

Responsive metal transcription factor 1 (MTF-1) also has an important role in coordinating cellular responses to metal homeostasis and oxidative stress. MTF-1 is a zinc-dependent transcription factor that stimulates the expression of metallothionein and zinc transporter-1 (ZnT-1) genes when the concentration of zinc increases. Zinc transporter ZnT-1 is located in cellular membranes and it allows the efflux of excess zinc from the cell. Through this function, it diminishes zinc toxicity in the cytosol. Furthermore, metallothionein also binds excess zinc ions in the cytosol. Thus, the induction of the expression of metallothionein and ZnT-1 is a mechanism that acts against elevated zinc levels [23,25].

MTF-1 also can protect cells from oxidative stress because this transcription factor is very sensitive to fluctuations in the redox cell status and promotes the expression of the target genes after stimulation. MTF-1 activates the expression of the Selenoprotein 1 (Sepw1) gene, which encodes an antioxidant glutathione-binding protein which scavenges free radicals [26]. Furthermore, this transcription factor regulates the immune response because it activates or suppresses the expression of genes encoding inflammatory cytokines [27].

It is important to note the ability of zinc to compete with iron and copper in the binding sites present in the cell membrane. Iron and copper ions can catalyze the production of radicals from lipid peroxides. In this way, the replacement of iron and copper by zinc could prevent the formation of highly reactive radicals because zinc is catalytically inert [11,28].

Linked to this, there is a close association between chronic low-grade inflammation and oxidative stress. Various pro-inflammatory transcription factors, including nuclear factor κB (NF-κB) and activator protein-1 (AP-1), can induce the production of ROS, causing the release of inflammatory cytokines. This in turn enhances oxidative stress, thus setting a vicious cycle [29]. While it is important that zinc also attenuates oxidative stress by acting as an anti-inflammatory nutrient, the precise mechanism remains unclear. Studies show that zinc regulates NF-κB transcription via the anti-inflammatory protein A20 and the receptor signaling pathway activated by peroxisome proliferator-α (PPAR-α) [30].

The study conducted by Prasad et al. [30] using cell cultures showed that cells with a high zinc concentration had increased expression of the A20 protein as well as reduced activation of the IκB kinase (IKK)-α/NF-κB signaling and pro-inflammatory cytokines. Bao et al. [31] found that zinc supplementation reduced the inflammatory molecules interleukin 6 (IL-6), TNF-α, chemoattractant protein monocytes (MCP-1), C-reactive protein, intercellular adhesion molecule 1 (ICAM-1), and E-selectin concentrations, and it increased the expression of PPAR-α and A20.

Research has shown the role of zinc deficiency in causing oxidative damage and, consequently, endothelial dysfunction. Zinc deficiency in endothelial cells enhances the inflammatory response mediated by cytokines and lipids, possibly via mechanisms associated with increased cellular oxidative stress. In contrast, zinc supplementation exerts a protective role against damage to the vascular system. The mechanisms by which zinc protects the blood vessels include the regulation of Nrf2, a transcription factor important for the expression of genes encoding antioxidant enzymes and for the induction and expression of metallothionein [32,33,34].

**Figure 1:**
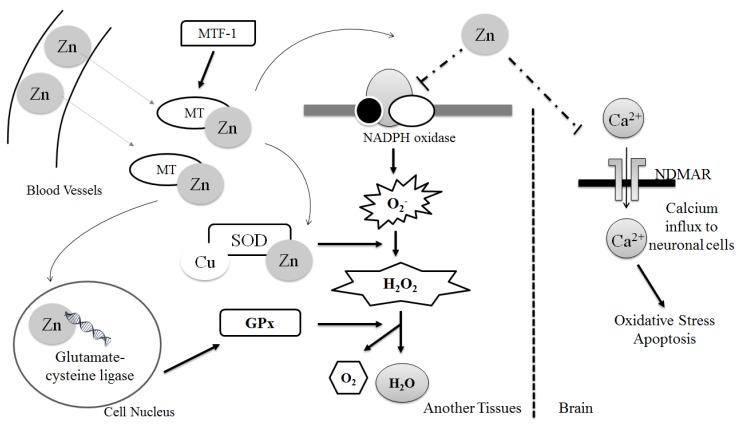
Zinc participation in antioxidant mechanisms. GPx: Glutathione peroxidase; MT: Metallothionein; MTF-1: Metal-responsive transcription factor 1; NADPH: nicotinamide adenine dinucleotide phosphate; NMDAR: N-methyl-d-aspartate receptor; SOD: superoxide dismutase enzyme; Zn: Zinc.

Studies have also demonstrated the role of zinc as an inhibitor of N-methyl-d-aspartate (NMDA) receptors, involved in the transport of calcium from the extracellular environment to the cytosol. Through this function, zinc deficiency promotes the activation of NMDA receptors and increases intracellular calcium concentration [35]. This increased intracellular calcium concentration promotes the release of substance P by neuronal cells. This neural mediator activates leukocytes and macrophages, which increases the release of inflammatory cytokines and the production of free radicals, contributing to the manifestation of oxidative stress [35]. Furthermore, in conditions where zinc is deficient, NADPH oxidase and nitric oxide synthase are activated, thereby favoring the production of reactive oxygen and nitrogen species [28] (Figure 1).

Zinc also improves insulin sensitivity and glycemic control because it contributes to reducing the synthesis of ROS under hyperglycemic conditions, thereby inhibiting the activation of oxidative stress pathways [12]. In this way, this micronutrient stimulates insulin secretion by the β-pancreatic cells, the phosphorylation of the β subunit of the insulin receptor and the activation of phosphatidylinositol protein 3-kinase and protein kinase B or Akt, important substrates for glucose uptake into cells. Thus, zinc favors glucose transport into the cells [36,37].

Recent research looking into the influence of zinc carrier proteins on cellular oxidative stress has shown the coordinated action of these carrier proteins in mitigating cellular damage. Liang et al. [38] demonstrated that zinc transporter ZnT-7 plays an important role in the survival of osteoblastic cell line MC3T3-E under oxidative stress conditions. The authors also found that hydrogen peroxide can induce the increase of intracellular zinc. ZnT-7 can protect these cells because this zinc transporter reduces the aggregation of free zinc ions and inhibits hydrogen peroxide–induced apoptosis. This function of ZnT-7 seems to involve the activation of tyrosine kinase proteins, which inhibit apoptosis by the phosphorylation of pro-apoptotic proteins when activated.

Moreover, oxidative stress appears to be capable of altering the expression of zinc carrier proteins. Sun et al. [39] demonstrated that chronic alcohol exposure reduces the expression of zinc transporters Zip-5 and Zip-14 in hepatocytes. Chronic ethanol exposure was also found to increase the expression of zinc transporters Zip-7 and ZnT-7 in hepatocytes, and this increase in protein expression was also observed in the treatment with hydrogen peroxide in vitro [39]. The altered expression of hepatic zinc transporters by ethanol exposure may disrupt the homeostasis of zinc in the liver. Zip-5 and Zip-14 are located on the plasma membrane of hepatocytes and, therefore, decreased expression of these transporters by ethanol exposure may be the cause of decreased zinc levels in the liver. Zip-7 and ZnT-7 are located on organelles, such as the endoplasmic reticulum and Golgi apparatus. Thus, altered expression of these zinc transporters may not only interfere with the homeostasis of zinc in organelles but may also lead to organelle dysfunction [39,40,41,42].

Chronic exposure to ethanol also significantly reduces zinc levels in the endoplasmic reticulum and mitochondria of isolated liver cells. Accordingly, treatment with zinc supplementation promotes the increased expression of Zip-8 and Zip-13, which transport zinc from the mitochondria and endoplasmic reticulum to the cytosol. Zinc supplementation also increases the expression of ZnT-4, which transports zinc from the cytosol to the mitochondria [43]. Zinc deficiency in these organelles appears to be associated with oxidative stress pathways, including the phosphorylation of initiation factor eukaryotic 4, the increased expression of C/EBP, the release of cytochrome c, and the insertion of Bax, a protein of the Bcl-2 family which activates cell death, with subsequent activation of caspase-3 and apoptosis [43].

Zinc is a potent inducer of metallothionein and is bound to metallothionein. Zinc is released from its complex with metallothionein in oxidative stress conditions. MTF-1, a zinc-dependent transcription factor, activates metallothionein gene expression, which can protect cells against oxidative stress. Zinc is a structural component of the enzyme superoxide dismutase, which promotes the conversion of two superoxide radicals to hydrogen peroxide and molecular oxygen. Zinc influences the expression of glutamate-cysteine ligase, an enzyme involved in the synthesis of glutathione, which acts directly on the neutralization of free radicals. Zinc also inhibits the NMDA receptors involved in the transport of calcium from the extracellular environment to the cytosol. Thus, zinc deficiency promotes the activation of NMDA receptors, which increase the intracellular concentration of calcium. In conditions where zinc is deficient, the NADPH oxidase enzymes and nitric oxide synthase are activated, favoring the production of reactive species of oxygen and nitrogen.

On the other hand, zinc also acts as a pro-oxidant when its concentration is either deficient or in excess and becomes pro-inflammatory and pro-apoptotic. Zinc excess also induces copper deficiency, which has been related to multiple adverse effects. These adverse effects include decreased expression of copper-dependent enzymes, such as superoxide dismutase and ceruloplasmin, which are important in antioxidant defense [44,45]. Moreover, zinc ions have a limited ability to bind to metallothionein, which is sensitive to situations of oxidative stress. This oxidative stress results in elevated concentrations of free zinc and induces a pro-oxidative status. A low concentration of zinc also leads to oxidative stress since this condition causes cell death and promotes the production of ROS [46,47].

## 3. Chronic Diseases

Research has shown that zinc status may be altered under pathophysiological conditions, such as diseases characterized by elevated oxidative stress, which impairs the control of these diseases. In particular, obese individuals may present with a deficiency of zinc, which aggravates the oxidative stress present in this disease [48,49]. A study conducted by Habib et al. [50] in obese individuals found high concentrations of malondialdehyde, a biomarker of lipid peroxidation, when compared with the control group. Associated with this, research has shown that low levels of glutathione and the reduction of superoxide dismutase activity may be due to zinc deficiencies observed in the patients studied.

Chronic hyperglycemia in type 2 diabetes mellitus has been associated with lipid peroxidation and oxidative damage in cells, impairing the antioxidant defense in type 2 diabetes patients. Lima et al. [12] showed reduced zinc concentrations in the plasma of type 2 diabetes patients. This is probably due to an elevated loss of this mineral in urine, resulting from hyperglycemia and polyuria. In addition, the authors also demonstrated an enhanced response to oxidative stress related to the appropriate zinc status and high superoxide dismutase activity in type 2 diabetic patients, the activity of which was influenced by the serum concentrations of insulin.

Zinc deficiency also can be found in chronic kidney disease, which has been attributed to reduced food intake and intestinal absorption of the mineral, interaction with calcium and iron and increased loss of minerals during dialysis. In this disease, zinc binds to proteins modified by uremia, such as albumin, thus aggravating its deficiency [51,52]. A study conducted by Magalhães et al. [52] showed reduced concentrations of zinc in patients with chronic kidney disease, which was correlated with the reduction in the activity of the enzyme superoxide dismutase and impairment of the antioxidant defense.

In the study by Guo and Wang [53], supplementation with 11 mg of zinc per day was able to increase the plasma concentration of this mineral and reduce the concentration of copper in patients undergoing hemodialysis for chronic renal failure. Zinc supplementation also reduced the plasma concentration of the oxidative product, malondialdehyde, and increased the activity of superoxide dismutase compared to the control patients.

The regulation of extracellular zinc concentrations is also extremely important for maintaining homeostasis of the neural network, because this mineral plays a role in both the physiology and pathophysiology of the brain [54]. In synapses, the main pool of zinc occurs in the presynaptic vesicles, where the free zinc is co-released with glutamate during neural activity and acts to suppress NMDA receptors in the synaptic cleft [55]. The inhibition of NMDA receptors reduces the release of substance P by neuronal cells, protecting organisms against oxidative stress [56].

Moreover, antioxidants and anti-carcinogenic mechanisms associated with zinc homeostasis appear to play an inhibitory role in the growth of neoplastic cells. Zinc acts in the protection against genomic instability and genetic mutations. In this sense, the superoxide dismutase is an anticarcinogenic enzyme. It inhibits the initiation, promotion, and progression phases in mammary carcinogenesis [57].

Furthermore, dietary zinc deficiency has also been linked to an increased risk for breast cancer [10,58]. Oxidative stress due to a lack of zinc leads to changes in the tissue of mammary glands and increased macrophage infiltration in this region, resulting in a toxic microenvironment. These mechanisms lead to a hyperaccumulation of the mineral, which is associated with increased expression of the alpha estrogen receptor (ERα), ductal changes in organization, and increased fibrosis in the mammary gland [58].

It is noteworthy that the expression of metallothionein is deregulated in breast cancer, which is correlated with resistance to chemotherapy and poor prognosis. Metallothionein participates in carcinogenesis via mechanisms that promote the development of tumor cells resistant to chemotherapy and radiation. High levels of metallothionein in cancer cells protect against damage by inhibiting free radicals, apoptosis and promoting cell proliferation. Thus, these functions support uncontrolled growth of these cancer cells. Moreover, the interaction of metallothionein with zinc ions is involved in the regulation of several transcription factors that contribute to carcinogenesis [59].

In diseases, such as epilepsy, schizophrenia, and Alzheimer’s and Parkinson’s diseases, the deleterious effects of zinc can also be observed. In these conditions, zinc is released in excess by presynaptic neurons and astrocytes, resulting in neuronal death via the activation of microglia, NADPH oxidase and the production of ROS in neurons [60,61,62]. Moreover, zinc is also involved in the apoptosis of brain cells induced by hypoxia or ischemia [63]. This study observed that treatment of cells in vitro and in animals with a zinc chelator, named N,N,N′,N′-Tetrakis(2-pyridylmethyl)ethylenediamine (TPEN), attenuated neurological deficit, reduced the rate of neuronal apoptosis and the cerebral infarct area, increased superoxide dismutase activity and reduced plasma concentrations of malondialdehyde and interleukin-6 (IL-6). Thus, zinc appears to play a role in preventing apoptosis in this situation, mainly via oxidative stress and inflammation pathways.

## 4. Conclusions

Research has still not fully elucidated the role of zinc in the molecular mechanisms involved in the pathophysiology of chronic diseases, such as obesity, diabetes and cancer. However, data in the literature show that a deficiency in zinc favors the manifestation of oxidative stress, promoting the development of these diseases. It is necessary to clarify the biochemical and molecular basis involved in this process. Therefore, further studies on the subject may elucidate the role of this nutrient in protecting against oxidative stress present in these diseases.
